# MR-guided radiotherapy in node-positive non-small cell lung cancer and severely limited pulmonary reserve: a report proposing a new clinical pathway for the management of high-risk patients

**DOI:** 10.1186/s13014-022-02011-8

**Published:** 2022-02-24

**Authors:** Chukwuka Eze, Elia Lombardo, Lukas Nierer, Yuqing Xiong, Maximilian Niyazi, Claus Belka, Farkhad Manapov, Stefanie Corradini

**Affiliations:** 1 Department of Radiation Oncology, University Hospital, LMU Munich, Marchioninistrasse 15, 81377 Munich, Germany; 2 German Cancer Consortium (DKTK), Partner Site Munich, Munich, Germany; 3 German Center for Lung Research (DZL), Comprehensive Pneumology Center Munich (CPC-M), Munich, Germany

**Keywords:** Hypofractionation, Image-guided radiotherapy, NSCLC, Pulmonary function, Thoracic radiotherapy

## Abstract

**Introduction:**

Online MR-guided radiotherapy (MRgRT) is a relatively novel advancement in the field of radiation oncology, ensuring superior soft-tissue visualisation, allowing for online plan adaptation to anatomical and functional interfractional changes and improved motion management. Platinum-based chemoradiation followed by durvalumab is the recommended treatment for stage IIB(N1)/III NSCLC. However, this is only the case for patients with favourable risk factors and sufficient pulmonary function and reserve.

**Methods:**

Herein, we present a technical report on tumour motion and breathing curve analyses of the first patient with node-positive stage IIB NSCLC and severely compromised pulmonary function and reserve [total lung capacity (TLC) 8.78L/132% predicted, residual volume (RV) 6.35L/271% predicted, vital capacity (VC) max 2.43L/58% predicted, FEV1 1.19L/38% predicted, DLCO-SB corrected for hemoglobin 2.76 mmol/min/kPa/30% predicted] treated in a prospective observational study with moderately hypofractionated MRgRT to a total dose of 48.0 Gy/16 daily fractions on the MRIdian system (Viewray Inc, Oakwood, USA).

**Results:**

Radiotherapy was well tolerated with no relevant toxicity. First follow-up imaging at 3 months post-radiotherapy showed a partial remission. The distinctive features of this case are the patient’s severely compromised pulmonary function and the first online MR-guided accelerated hypofractionated radiotherapy treatment for primary node-positive NSCLC.

**Conclusions:**

This technical report describes the first patient treated in a prospective observational study evaluating the feasibility of this relatively novel technology in stage IIB(N1)/III disease, proposing a clinical pathway for the management of high-risk patients.

**Supplementary Information:**

The online version contains supplementary material available at 10.1186/s13014-022-02011-8.

## Introduction

Chemoradiation (CRT) followed by consolidation with durvalumab is the recommended treatment strategy for the management of stage IIB (N1)/III non-small cell lung cancer (NSCLC) [1]. However, in patients with poor prognostic factors, there is no consensus on the optimal treatment regimen, and patients are often referred to palliative radiotherapy alone or best supportive care. Our group has previously demonstrated the feasibility of PET-based accelerated hypofractionated radiotherapy (AHRT) on a conventional linear accelerator (LINAC) in this distinct patient cohort with encouraging outcome [2, 3].

Online MR-guided radiotherapy is a promising novel advancement in the field of radiation oncology allowing for superior real-time tumour visualisation, plan adaptation to anatomical changes and improved motion management [4–6]. Based on our previous experience, the Ludwig Maximilian University of Munich ethics committee approved a protocol (reference number: 20-793) for MRgRT in high-risk patients. In theory, due to online soft-tissue visualisation and gating capabilities, there is less uncertainty when delivering radiotherapy which subsequently could support dose-escalation strategies. Herein, we present a comprehensive technical report of the first patient treated with this protocol.

## Case presentation

A 60-year-old male cachectic patient (172 cm, 45 kg; BMI: 15.2 kg/m^2^) presented to a specialised clinic in the treatment of respiratory diseases in 11/2020 following detection of a suspicious lung mass in SII of the right lung and an adjacent enlarged 15 mm lymph node (LN) in position 11R on follow-up CT for previous oropharyngeal cancer. The patient had an Eastern Cooperative Oncology Group performance status (ECOG-PS) of 2 and was an active smoker (40 pack years). He presented with chronic shortness of breath and persistent cough. His medical history included previous oropharyngeal cancer with first diagnosis in 11/2016. He underwent transoral laser surgery (pT2 pN0 cM0 R1) and adjuvant CRT to a total dose of 66.0 Gy ex domo. Other secondary diagnoses included COPD GOLD 3; group B, insulin-dependent diabetes mellitus, recurrent acute pancreatitis. A pulmonary function test (PFT) on admission, revealed the following results: total lung capacity (TLC) 8.78L/132% predicted, residual volume (RV) 6.35L/271% predicted, vital capacity (VC) max 2.43L/58% predicted, forced expiratory volume in 1 s (FEV1) 1.19L/38% predicted, single breath diffusing capacity of lung for carbon monoxide corrected for haemoglobin (DLCOcSB) 2.76 mmol/min/kPa/30% predicted. In addition, a blood gas analysis in room air was performed: pH: 7.40, pO2: 67 mmHg, pCO2: 44 mmHg, BE: 2.40 mmol/l, STB: 26.1 mmol/l, SaO2: 95%.

**Fig. 1:**
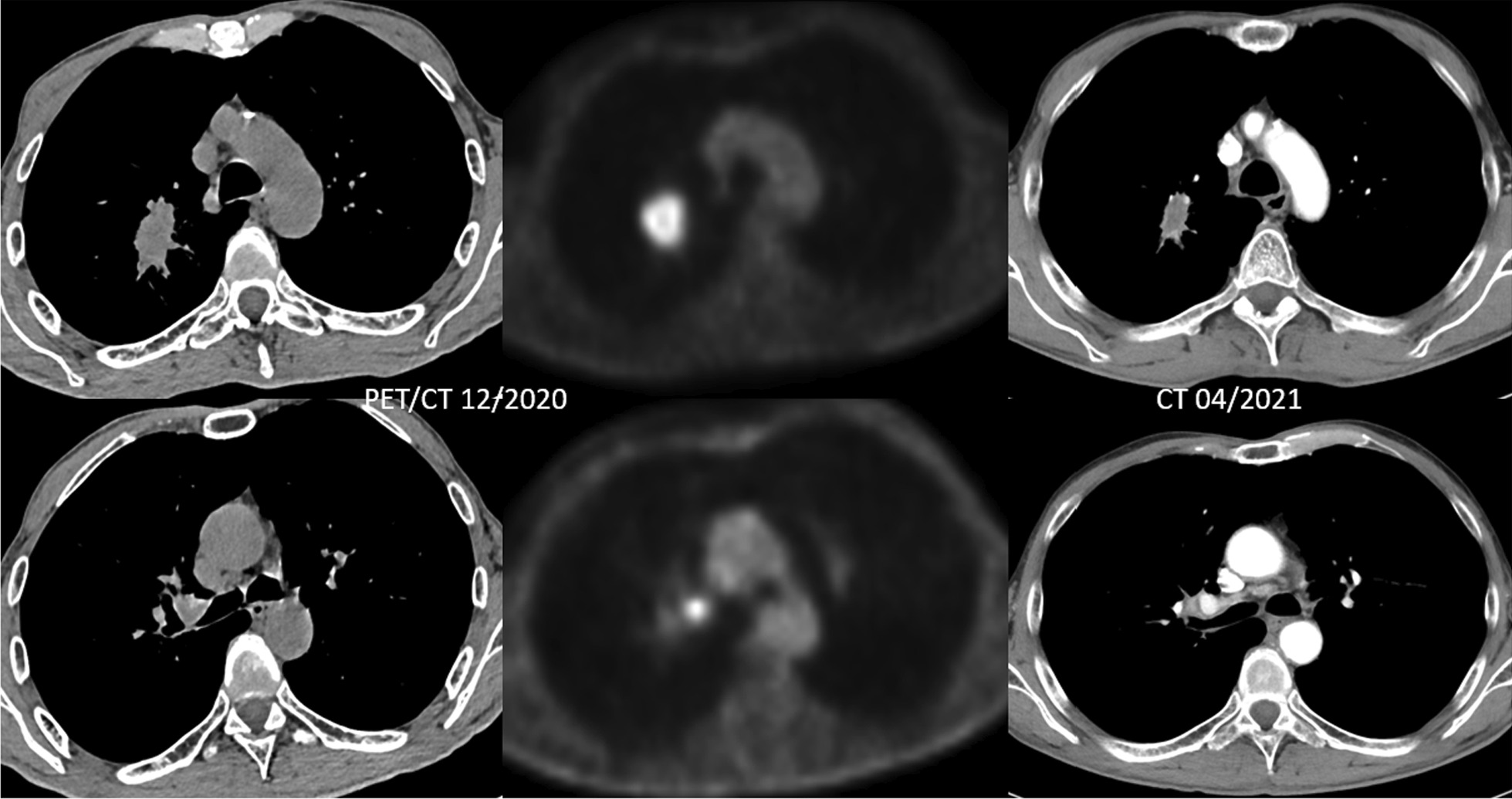
PET/CT at initial diagnosis 12/2020; note enhanced FDG-uptake in the primary tumour (SUVmax 7) and LN pos. 11R (SUVmax 4) and Follow-up CT 3 months post-MRgRT in 04/2021 demonstrating a partial remission

Bronchoscopy and transbronchial biopsy (TBB) with endobronchial ultrasound-guided transbronchial needle aspiration (EBUS-TBNA) 7/11R was performed with the histopathological diagnosis of CK7, Napsin A pos.; CDX2, TTF-1, p40 neg. primary adenocarcinoma of the lung; PD-L1 negative/Cologne Score: 0 (E1L3N). His insurance company did not cover further molecular pathology testing in the primary setting. PET/CT confirmed a 3.2 × 2.5 × 3.6 cm FDG-avid tumour (SUVmax 7) in the right upper lobe with moderate FDG-uptake in a LN at position 11R (SUVmax 4) (Fig. 1). MRI of the brain was inconspicuous, hence stage IIB-cT2a cN1 cM0 (UICC 8th edition) disease. Following discussion at the multidisciplinary tumour board (MTB), the patient was referred for moderately hypofractionated radiotherapy 48.0 Gy in 16 fractions.

### Treatment planning and delivery

**Fig. 2:**
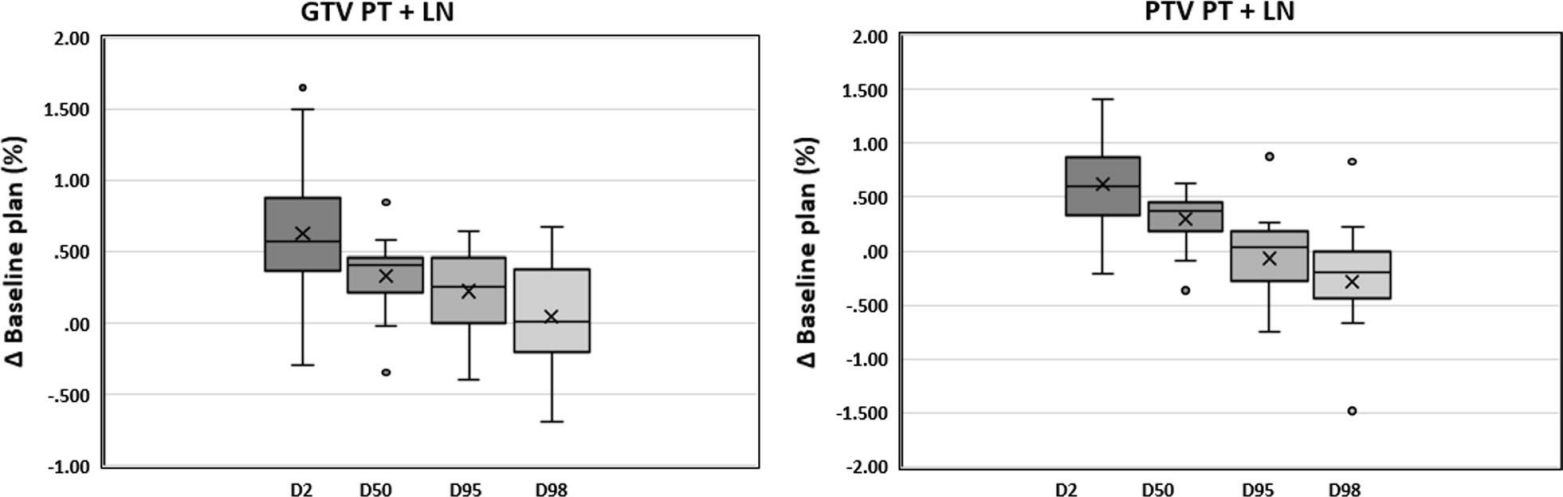
Box-plot of percentage change in D2, D50, D95 and D98 for all 16 fractions compared to the baseline plan

**Fig. 3:**
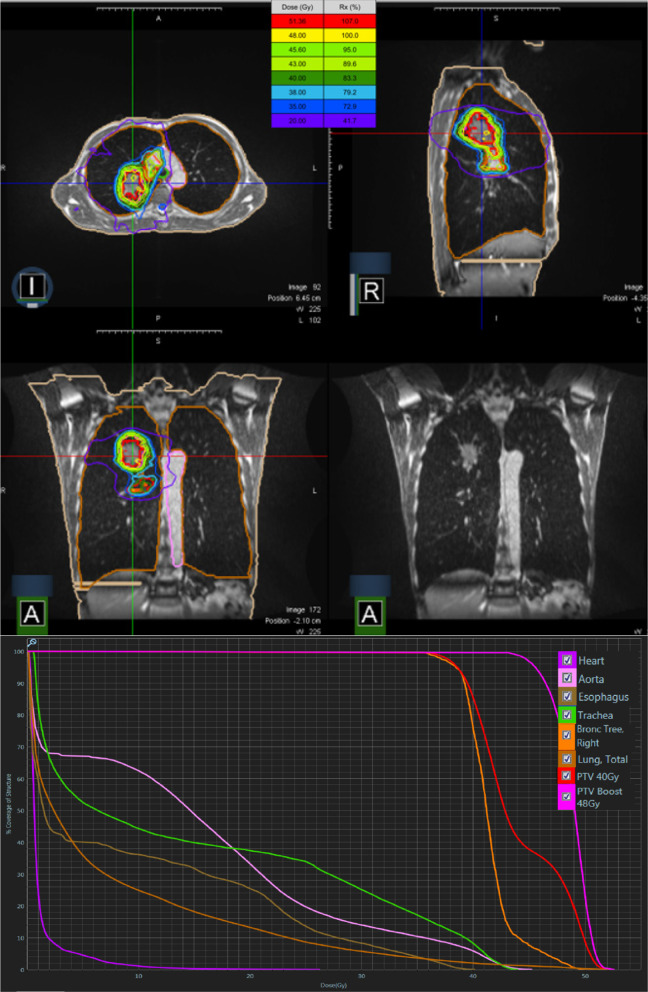
Isodose distribution for representative slices in all main planes and the corresponding dose-volume histogram with the green colour depicting 95% (45.6 Gy) of the prescribed dose to the PTV Boost and the cyan colour depicting 95% (38.0 Gy) of the prescribed dose to the PTV 40 Gy

Briefly, the treatment was delivered on one of two commercially available MR-guided linear accelerator systems, the MRIdian system (Viewray Inc, Oakwood, USA) [7, 8]. The patient underwent MRI simulation in inspiration breath-hold (BH) and supine position with arms above the head using a dedicated positioning device (WingSTEP, IT-V, Innsbruck, Austria). Thereafter, a standard planning CT using the same patient positioning and BH level was conducted to obtain electron density information. Image datasets were then co-registered using the deformable registration algorithm of the integrated MRIdian treatment planning system. The target volume and OARs were contoured on the 3D MR simulation scan. An isotropic gross tumour volume (GTV) expansion of 5 mm was used to generate the planning target volume (PTV) primary tumour + LN. Due to multiple suspicious mediastinal LNs in pos. 2R, regional nodal irradiation of stations 10R/4R/2R was performed. GTV primary tumour + LN was 35.12 cc and PTV primary tumour + LN was 80.41 cc. Radiotherapy was delivered in a non-adaptive manner with the baseline plan, due to limited interfractional anatomical changes. This was also confirmed by the minimal dosimetric changes of the GTV/PTV dose coverage over the course of the treatment (Fig. 2). The relevant dosimetric parameters of organs at risk (OARs) for the baseline plan are provided in Table 1. During dose delivery, continuous, real-time 2D cine MRI in a single sagittal plane was used to monitor the target volume. For this purpose, a gating boundary contour was defined by adding an isotropic margin of 3 mm to the GTV and a pre-defined threshold region of interest percentage (ROI%) was set at 5%, allowing a percentage of the GTV to be outside the gating boundary before a beam-hold is triggered. The beam was gated automatically by the gating functionality of the system through a deformable registration-based tracking algorithm. The total delivery time for all fractions was 2.35 h and total “beam-on” time for all fractions was 27.78 min (Table 2). The dose covering 98% (D98), 50% (D50) of the GTV and PTV primary tumour was 47.6 Gy, 49.70 Gy and 44.6 Gy, 49.1 Gy, respectively. The dose to 2% (D2) of the GTV and PTV primary tumour was 51.5 Gy and 51.3 Gy. The isodose distribution for representative slices in all planes and the corresponding dose-volume histogram (DVH) is depicted in Fig. 3. In addition, a cine movie extract of the first fraction is provided in the Additional file 1: Video S1.

**Table 1:** Relevant organs at risk dosimetric parameters of the baseline plan; *MLD* mean lung dose, relative organ volume receiving ≥ 5 Gy (V5), ≥ 10 Gy (V10), ≥ 20 Gy (V20)

Lung, total			
V5 (%)	V10 (%)	V20 (%)	MLD (Gy)
36.0	24.9	13.3	7.4
Lung, right			
V5 (%)	V10 (%)	V20 (%)	MLD (Gy)
45.6	40.3	24.9	11.0
Lung, left			
V5 (%)	V10 (%)	V20 (%)	MLD (Gy)
25.4	8.2	0.6	3.6
Esophagus			
Mean (Gy)	V5 (%)	V10 (%)	V20 (%)
9.5	40.2	36.2	25.5
Heart			
Mean (Gy)	V5 (%)	V10 (%)	V20 (%)
1.2	4.6	1.1	0.1

**Table 2:** Treatment delivery times and resulting duty cycle efficiencies for each fraction

	Delivery time (mins)	Beam-on time (mins)	Duty cycle efficiency (%)
Fr 1	8.48	1.71	20.20
Fr 2	9.68	1.73	17.86
Fr 3	8.86	1.71	19.32
Fr 4	8.49	1.69	19.88
Fr 5	9.04	1.72	18.99
Fr 6	8.86	1.73	19.46
Fr 7	9.10	1.71	18.83
Fr 8	8.84	1.72	19.46
Fr 9	8.40	1.72	20.45
Fr 10	9.86	1.73	17.58
Fr 11	8.50	1.74	20.45
Fr 12	9.18	1.79	19.48
Fr 13	8.47	1.77	20.96
Fr 14	8.45	1.8	21.24
Fr 15	8.38	1.76	20.98
Fr 16	8.43	1.75	20.76

### Tumour motion and gating analysis

**Fig. 4:**
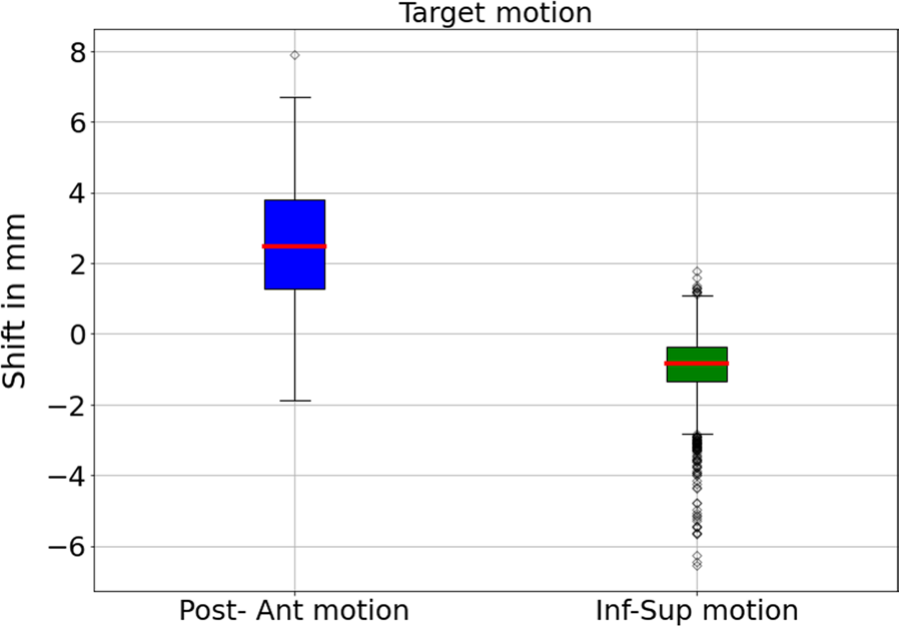
Box-plot of the primary tumour motion for fraction 1. We calculated the median and inter-quartile range (IQR) amplitudes of the extracted target centroid positions relative to the centroid position of the boundary contour. For the first fraction, the target posterior-anterior motion was 2.48/2.53 mm (median/IQR) and -0.85/0.98 mm (median/IQR) for the inferior-superior motion. Box-plots for every fraction are provided in the Additional file 2

Tumour centroid positions were extracted from the cine videos using an in-house developed code. Using the centroid positions, we then analysed the primary tumour motion in the posterior-anterior and inferior-superior directions for all fractions as shown exemplarily for the first fraction (Fig. 4) and all fractions (Additional file 2: S2-1 to S2-15).

In total, 2.35 h of real-time cine images were analysed. Using the beam-on status displayed in the cine videos, we computed the total beam-on time for all fractions, which amounted to 27.78 min. Additionally, we calculated the duty cycle efficiency for each fraction, defined as the number of “beam-on” frames divided by the total number of MR cine frames acquired during that fraction, including frames acquired during multileaf collimator motion and gantry rotation according to Finazzi et al. [9]. Information about the imaging pauses was also extracted from the cine videos. The data for all fractions is provided in Table 2.

**Fig. 5:**
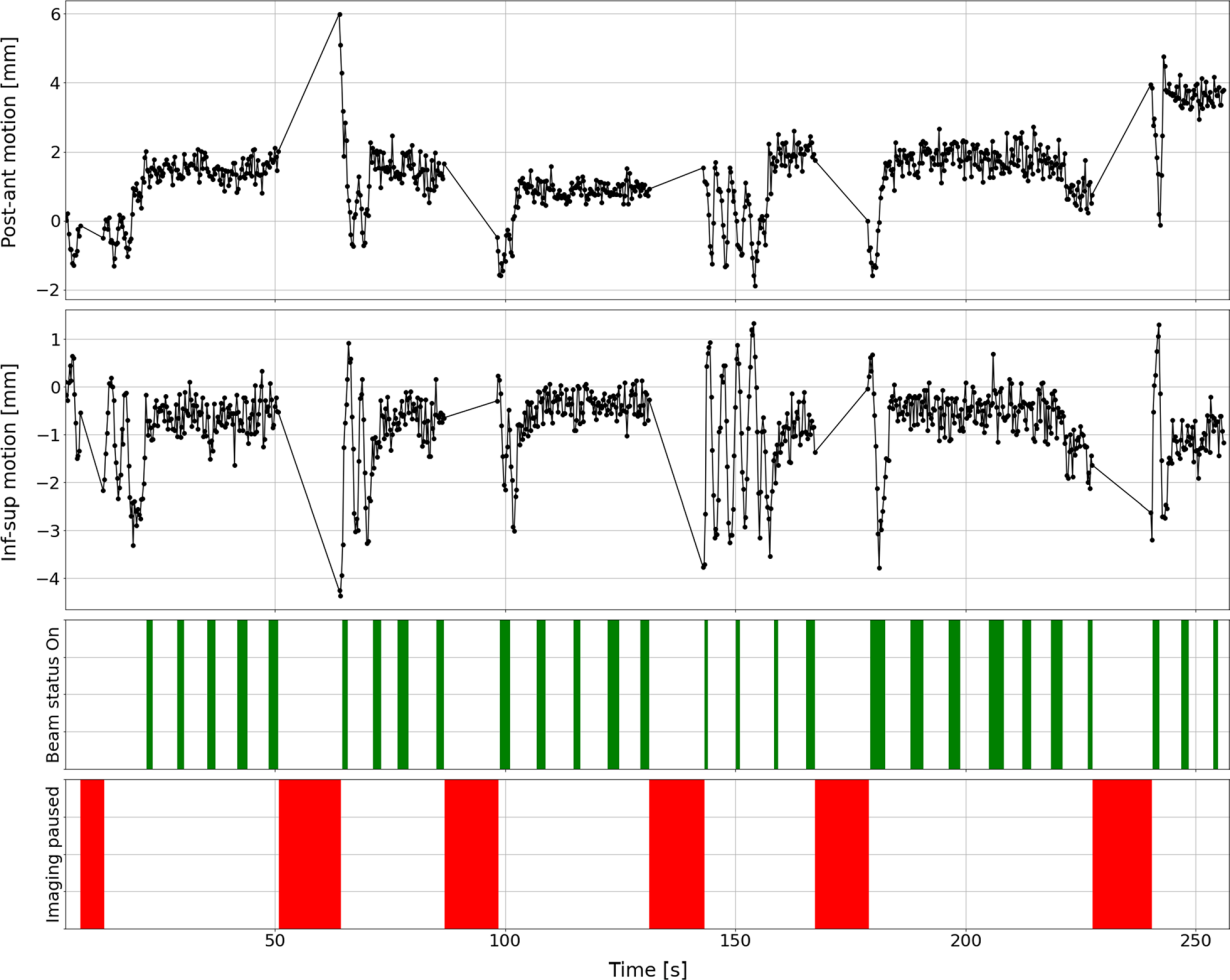
Primary tumour motion in posterior-anterior and inferior-superior directions for the first few minutes of fraction 1; “Beam-on” time intervals are denoted by the green bars while time intervals where the cine imaging was paused are denoted by the red bars. During gantry rotation, acquisition of cine imaging was paused hence the patient breathes freely

The Primary tumour motion in posterior-anterior and inferior-superior directions for the first few minutes of fraction 1 is depicted in Fig. 5; “Beam-on” time intervals are denoted by the green bars while time intervals where the cine imaging was paused are denoted by the red bars. During gantry rotation, acquisition of cine imaging was paused hence the patient breathes freely.

### Patient outcome

Treatment was well tolerated with no relevant (grade ≥ 2) toxicity. On the first follow-up CT scan 3 months post-MRgRT (04/2021), a decrease in the target lesion dimensions from 3.2 × 2.5 × 3.6 cm to 2.7 × 1.2 × 2.3 cm was noted, hence partial remission per RECIST 1.1 criteria (Fig. 1). The patient remained an active smoker and last visited with his pulmonologist early October 2021 in reduced general condition (age-adjusted Charlson Comorbidity Index: 7; BMI: 14 kg/m^2^) and died late October 2021 (8.8 months post-MRgRT and 11.2 months from initial diagnosis) with the cause of death unclear but possibly related to pulmonary disease.

## Discussion

To the best of our knowledge, the present case is the first report on MR-guided radiotherapy for node-positive NSCLC. Importantly, this patient had reduced PS and limited pulmonary function and reserve, which precluded concurrent CRT. MRgRT was feasible with no significant overlap with the previous radiotherapy in the head-and-neck region. On first follow-up post-RT, the patient presented in stable condition with a partial remission (Fig. 1).

Regarding the concept of moderate hypofractionation, Amini et al. [10] reported comparable oncologic outcome in 300 stage III patients treated with 45.0 Gy/15 vs. ≥ 60.0 Gy/2.0 Gy fractions, although patients in the hypofractionation arm initially had worse prognostic factors. Similarly, we previously published encouraging survival data of prospectively enrolled patients with poor prognostic factors and limited pulmonary reserve (FEV1 ≤ 1.0 L and/or DLCO-SB ≤ 40% predicted and/or on long-term oxygen therapy) treated on a conventional linear accelerator [3]. Hence, due to the severely compromised lung function, a moderate hypofractionation schedule was chosen to account for normal lung tissue dose exposure. Furthermore, the application of MRgRT in this setting has its inherent advantages. Due to the incorporation of a 0.35 T MR scanner, superior soft tissue contrast aids in more accurate delineation of central tumours in close proximity to mediastinal structures [4]. Online MRgRT ensures daily plan adaptation in particular in challenging cases e.g. with accompanying atelectasis and subsequent resolution during treatment thereby mitigating an overdose to organs at risk [11]. In the case of favourable tumour response, online adaptation to morphological changes to the tumour are readily feasible. Importantly, online MRgRT enables real-time tumour monitoring during treatment and motion management by means of gating/tracking strategies thus eliminating the need for larger margins or internal target volume (ITV) concepts to account for tumour mobility. To further unlock new capabilities of this novel technology, functional imaging including diffusion-weighted imaging facilitates response assessment during and after primary treatment [12]. Therefore, promoting adaptive and dose-escalation strategies based on biological response similar to concepts employed with PET imaging [13, 14]. All these aspects in comparison to treatments on a conventional LINAC ensure that there is less uncertainty when delivering radiotherapy and support isotoxic dose escalation strategies. In conjunction with our previous experience, the present analysis provides a clinical pathway for the management of these high-risk patients.

Currently, an ongoing phase II study is exploring the feasibility of MR-guided hypofractionated adaptive radiation therapy of 60.0 Gy/15 fractions with concurrent chemotherapy and consolidation durvalumab in unresectable stages IIB-IIIC NSCLC (NCT03916419). Furthermore, the combination of radiotherapy and immune-checkpoint inhibitors can potentiate anti-tumour response and improve patient outcome [15]. A number of trials are exploring conventional/hypofractionated radiotherapy in node-positive NSCLC with PD-L1 inhibition in (borderline) patients [SWOG S1933 (NCT04310020), ARCHON-1 study (NCT03801902), TRADE-hypo trial (NCT04351256) and the SPIRAL-RT study (JMA-IIA00434)].

## Conclusion

We report the first case of a patient with stage IIB(N1) NSCLC and severely limited pulmonary function and reserve treated with MRgRT, hereby proposing a clinical pathway for the management of high-risk patients. We are currently recruiting patients to our MRgRT protocol.

## Supplementary Information

**Additional file 1**: Extract of gated MRgRT delivery for the first fraction of treatment. The gating target (GTV; green contour) and the gating boundary (red contour) are displayed.

**Additional file 2**: Box-plot of the primary tumour motion in posterior-anterior and inferior-superior directions for fractions 2-16.

## Data Availability

Substantial data generated or analysed during this study are included in this published article [and its supplementary information files]. Further datasets during and/or analysed during the current study are available from the corresponding author on reasonable request.
