# Supplementary figures and images for: MR-guided radiotherapy in node-positive non-small cell lung cancer and severely limited pulmonary reserve: a report proposing a new clinical pathway for the management of high-risk patients
Source: Radiat Oncol. 2022 Feb 24;17:43. doi: 10.1186/s13014-022-02011-8 (PMC8876180; doi:10.1186/s13014-022-02011-8)

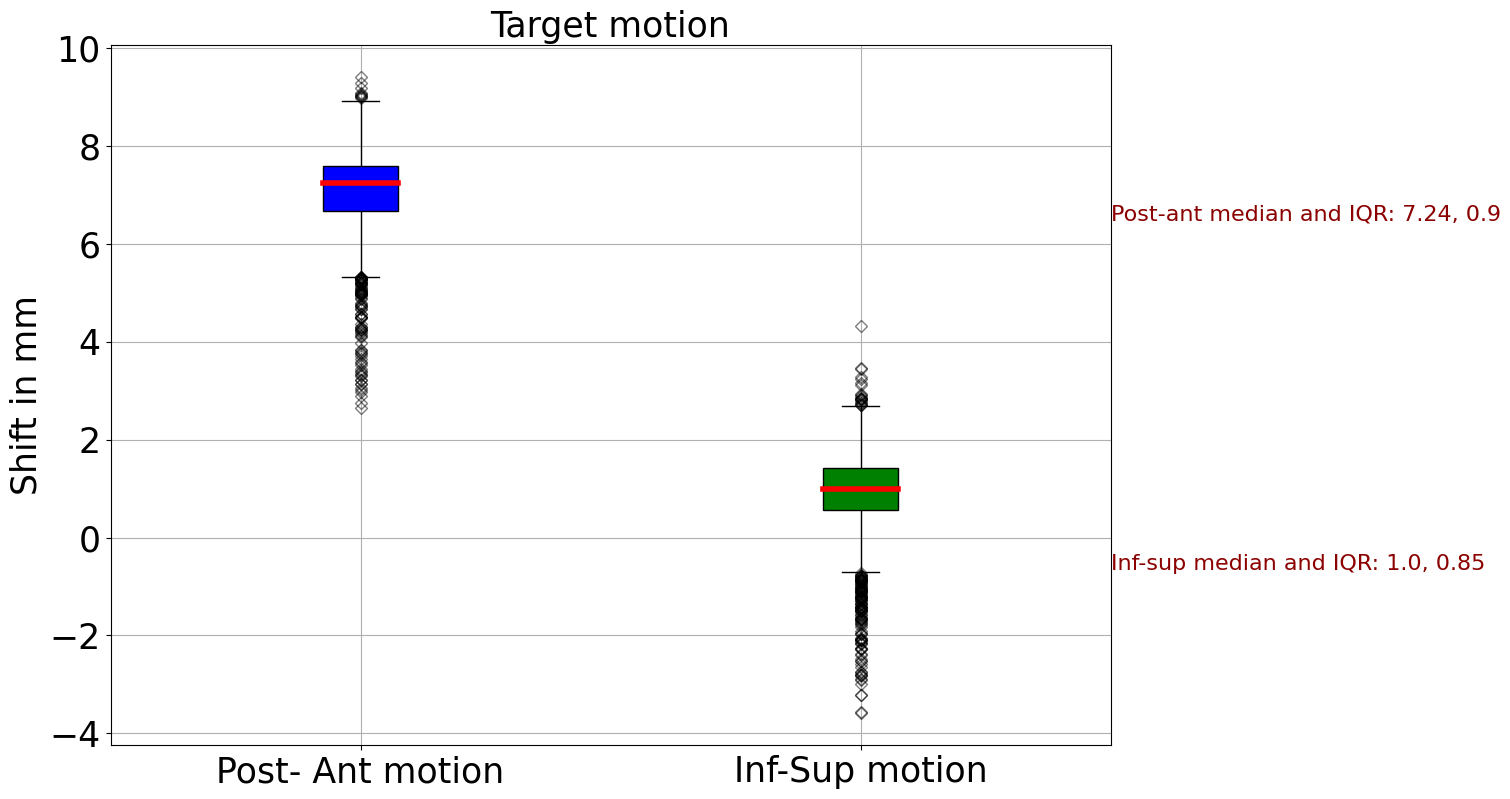

Supplement: Supplementary file 2 — Additional file 2: Box-plot of the primary tumour motion in posterior-anterior and inferior-superior directions for fractions 2-16. [file 13014_2022_2011_MOESM2_ESM.zip › 13014_2022_2011_MOESM2_ESM/S2-01.png]

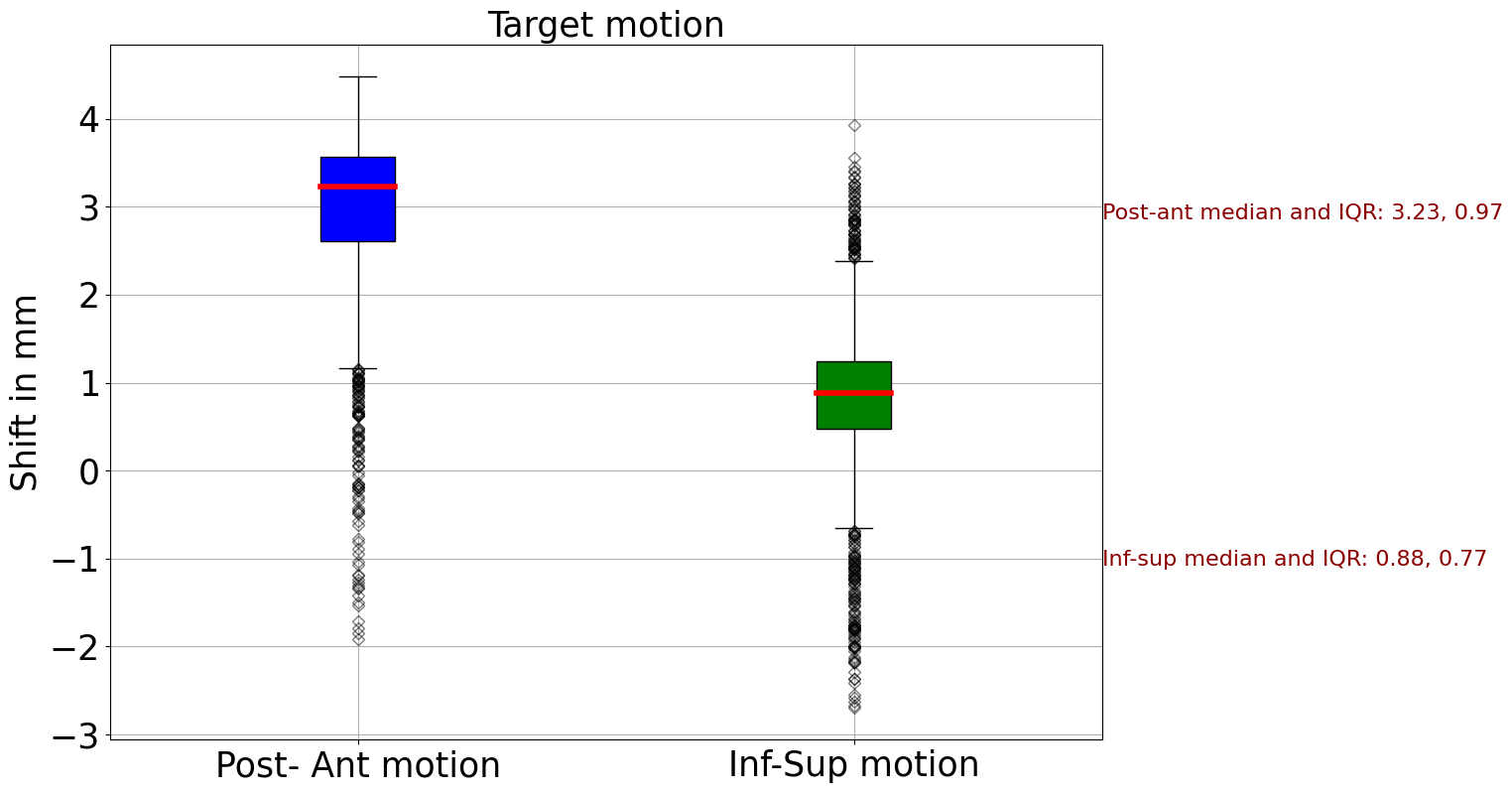

Supplement: Supplementary file 2 — Additional file 2: Box-plot of the primary tumour motion in posterior-anterior and inferior-superior directions for fractions 2-16. [file 13014_2022_2011_MOESM2_ESM.zip › 13014_2022_2011_MOESM2_ESM/S2-02.png]

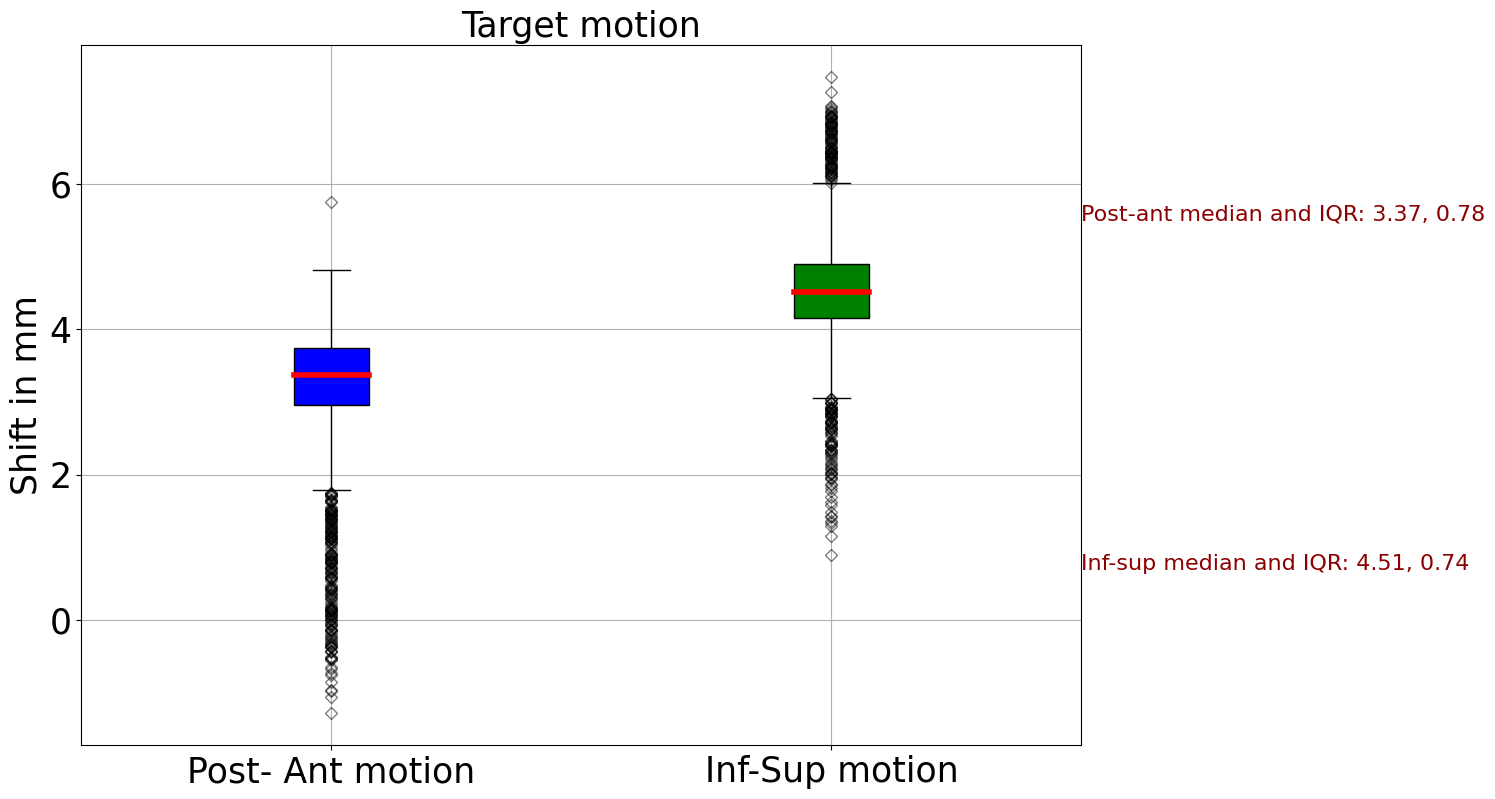

Supplement: Supplementary file 2 — Additional file 2: Box-plot of the primary tumour motion in posterior-anterior and inferior-superior directions for fractions 2-16. [file 13014_2022_2011_MOESM2_ESM.zip › 13014_2022_2011_MOESM2_ESM/S2-03.png]

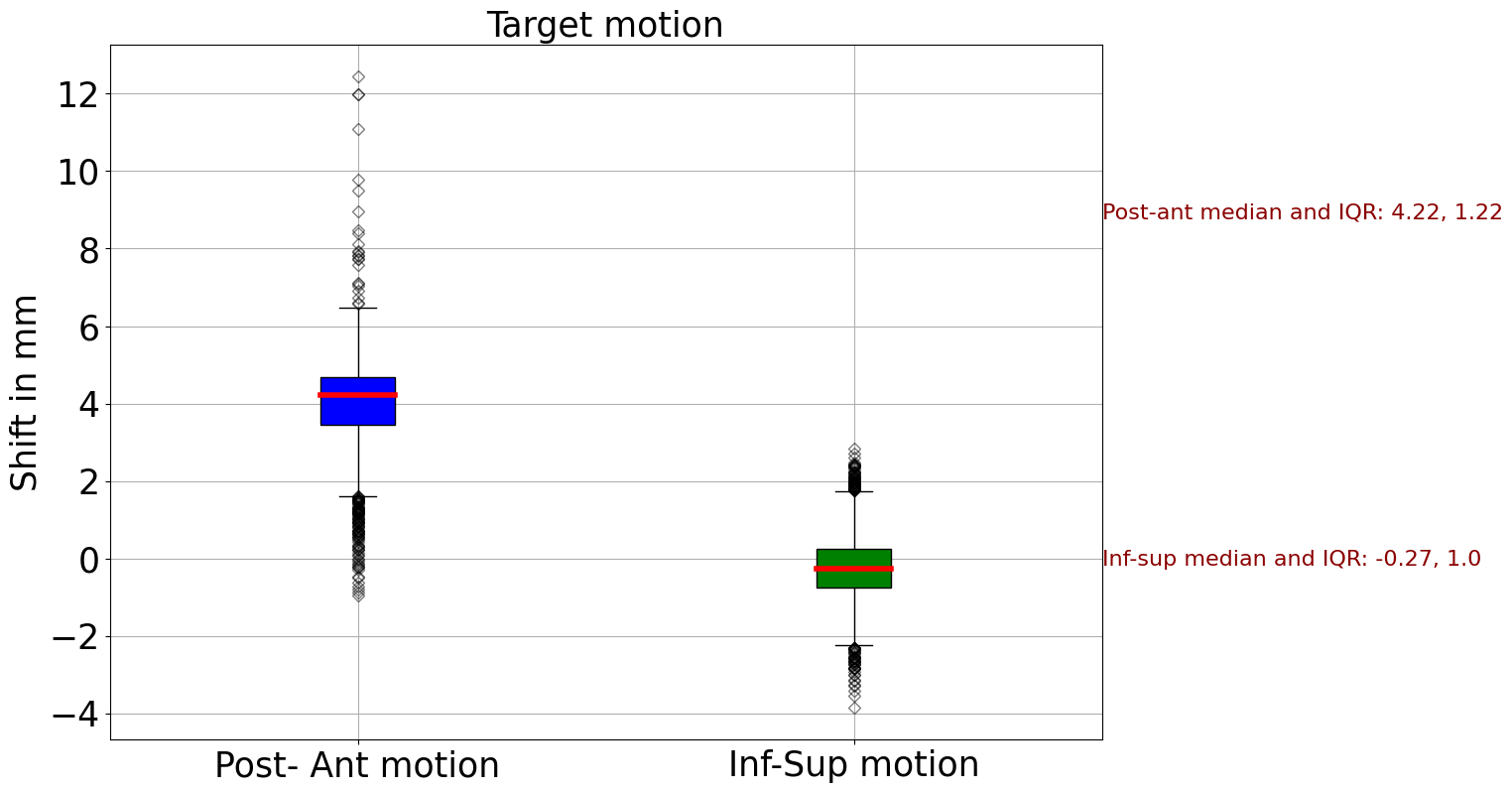

Supplement: Supplementary file 2 — Additional file 2: Box-plot of the primary tumour motion in posterior-anterior and inferior-superior directions for fractions 2-16. [file 13014_2022_2011_MOESM2_ESM.zip › 13014_2022_2011_MOESM2_ESM/S2-04.png]

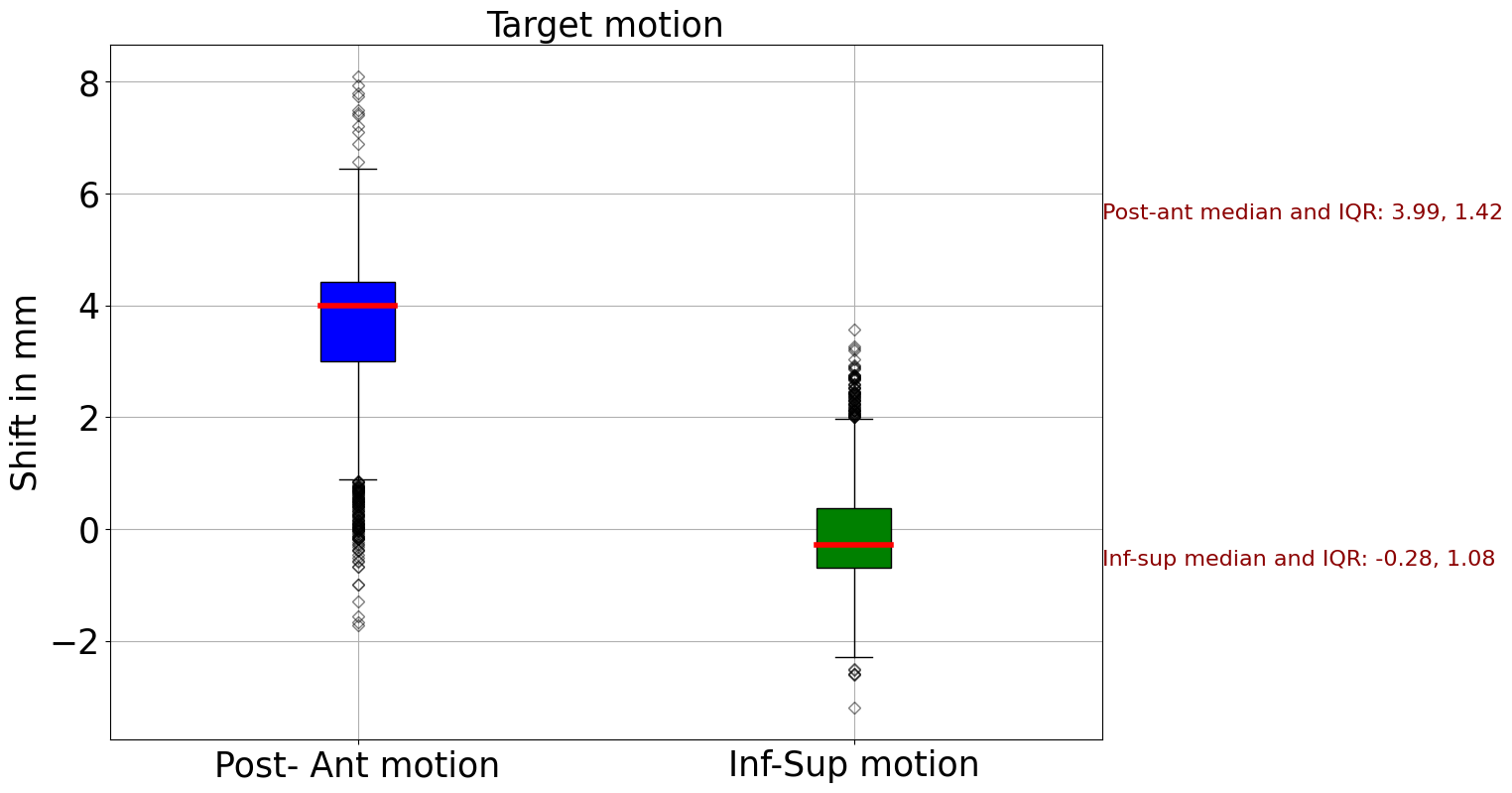

Supplement: Supplementary file 2 — Additional file 2: Box-plot of the primary tumour motion in posterior-anterior and inferior-superior directions for fractions 2-16. [file 13014_2022_2011_MOESM2_ESM.zip › 13014_2022_2011_MOESM2_ESM/S2-05.png]

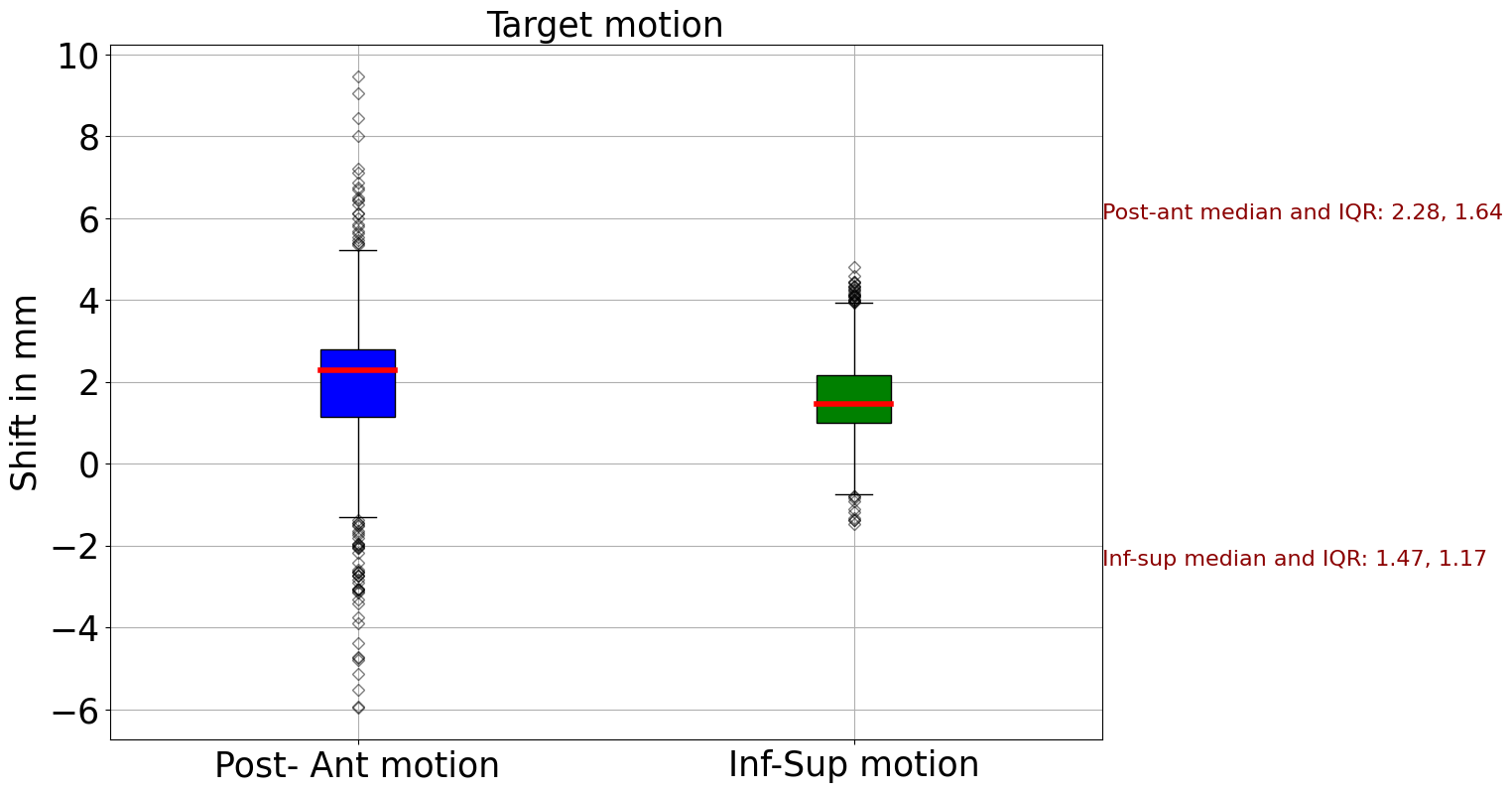

Supplement: Supplementary file 2 — Additional file 2: Box-plot of the primary tumour motion in posterior-anterior and inferior-superior directions for fractions 2-16. [file 13014_2022_2011_MOESM2_ESM.zip › 13014_2022_2011_MOESM2_ESM/S2-06.png]

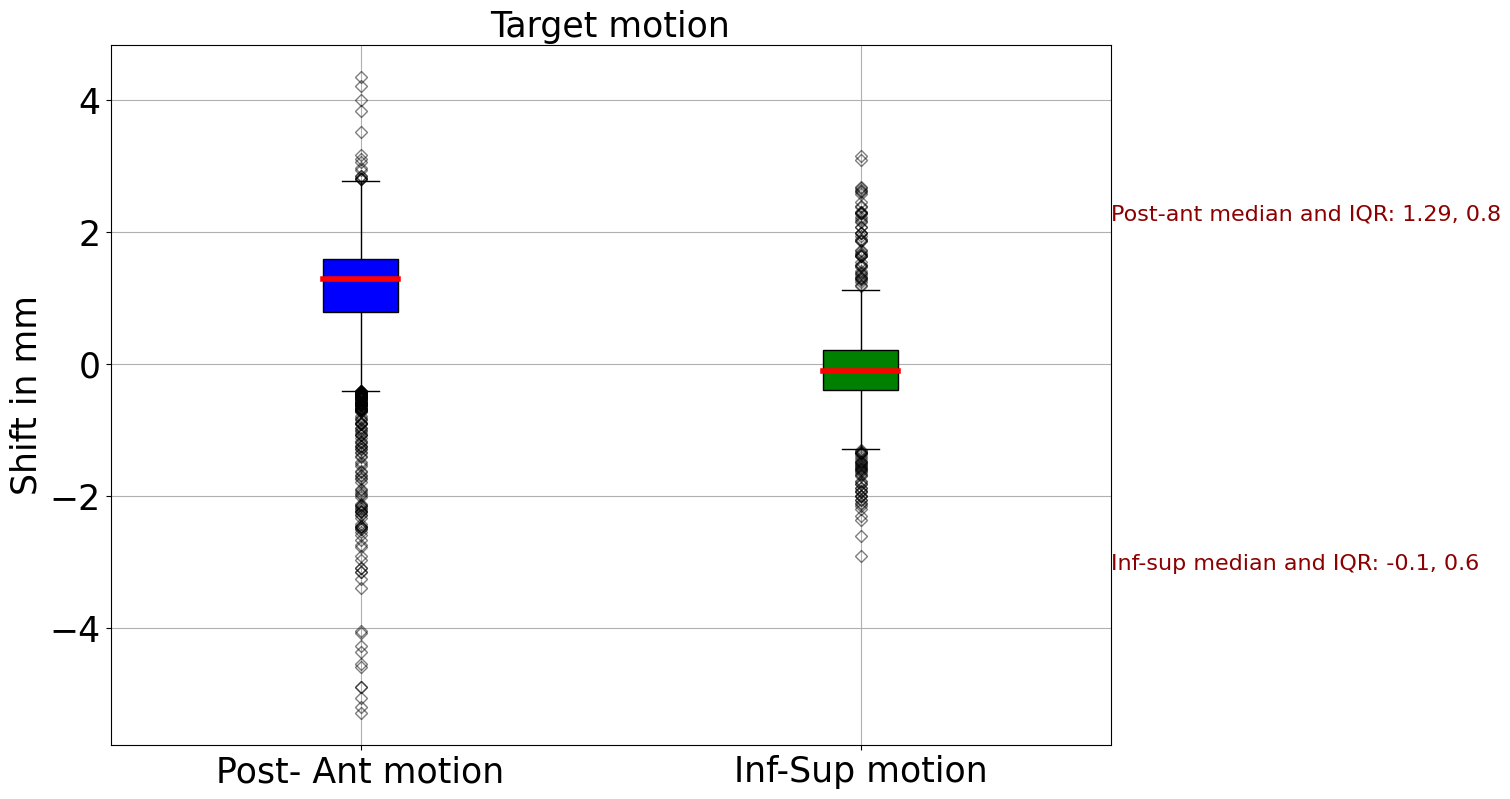

Supplement: Supplementary file 2 — Additional file 2: Box-plot of the primary tumour motion in posterior-anterior and inferior-superior directions for fractions 2-16. [file 13014_2022_2011_MOESM2_ESM.zip › 13014_2022_2011_MOESM2_ESM/S2-07.png]

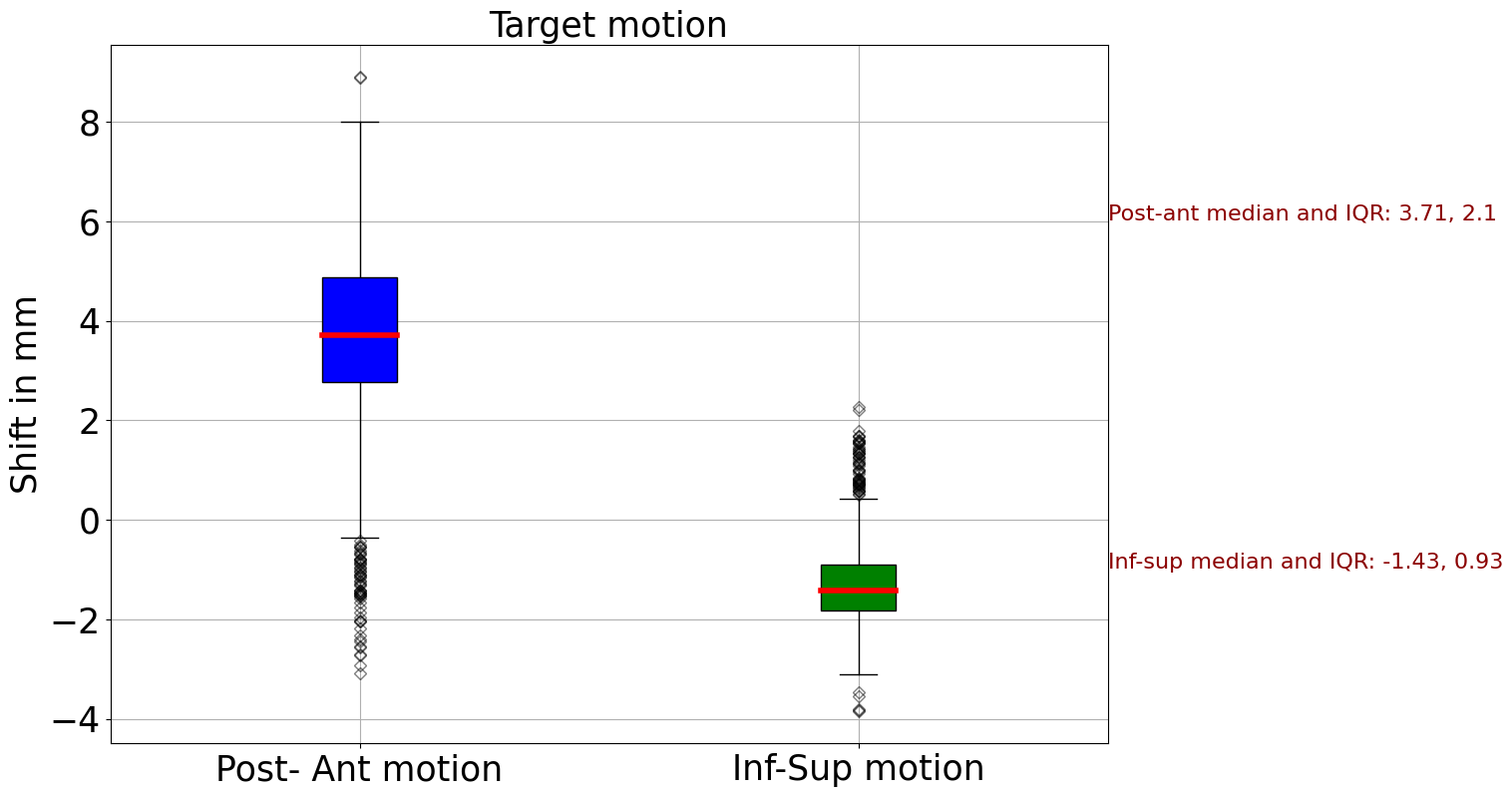

Supplement: Supplementary file 2 — Additional file 2: Box-plot of the primary tumour motion in posterior-anterior and inferior-superior directions for fractions 2-16. [file 13014_2022_2011_MOESM2_ESM.zip › 13014_2022_2011_MOESM2_ESM/S2-08.png]

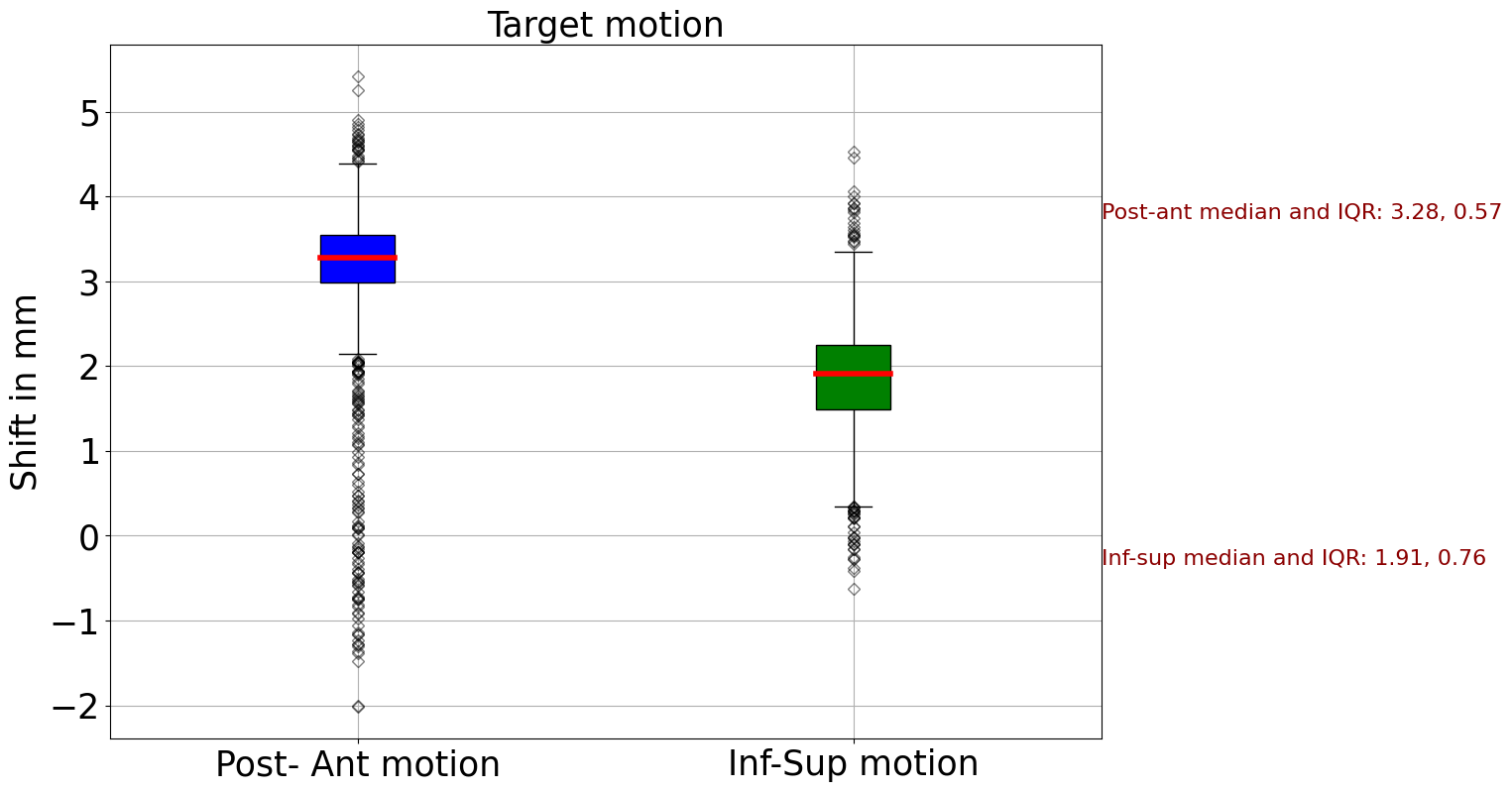

Supplement: Supplementary file 2 — Additional file 2: Box-plot of the primary tumour motion in posterior-anterior and inferior-superior directions for fractions 2-16. [file 13014_2022_2011_MOESM2_ESM.zip › 13014_2022_2011_MOESM2_ESM/S2-09.png]

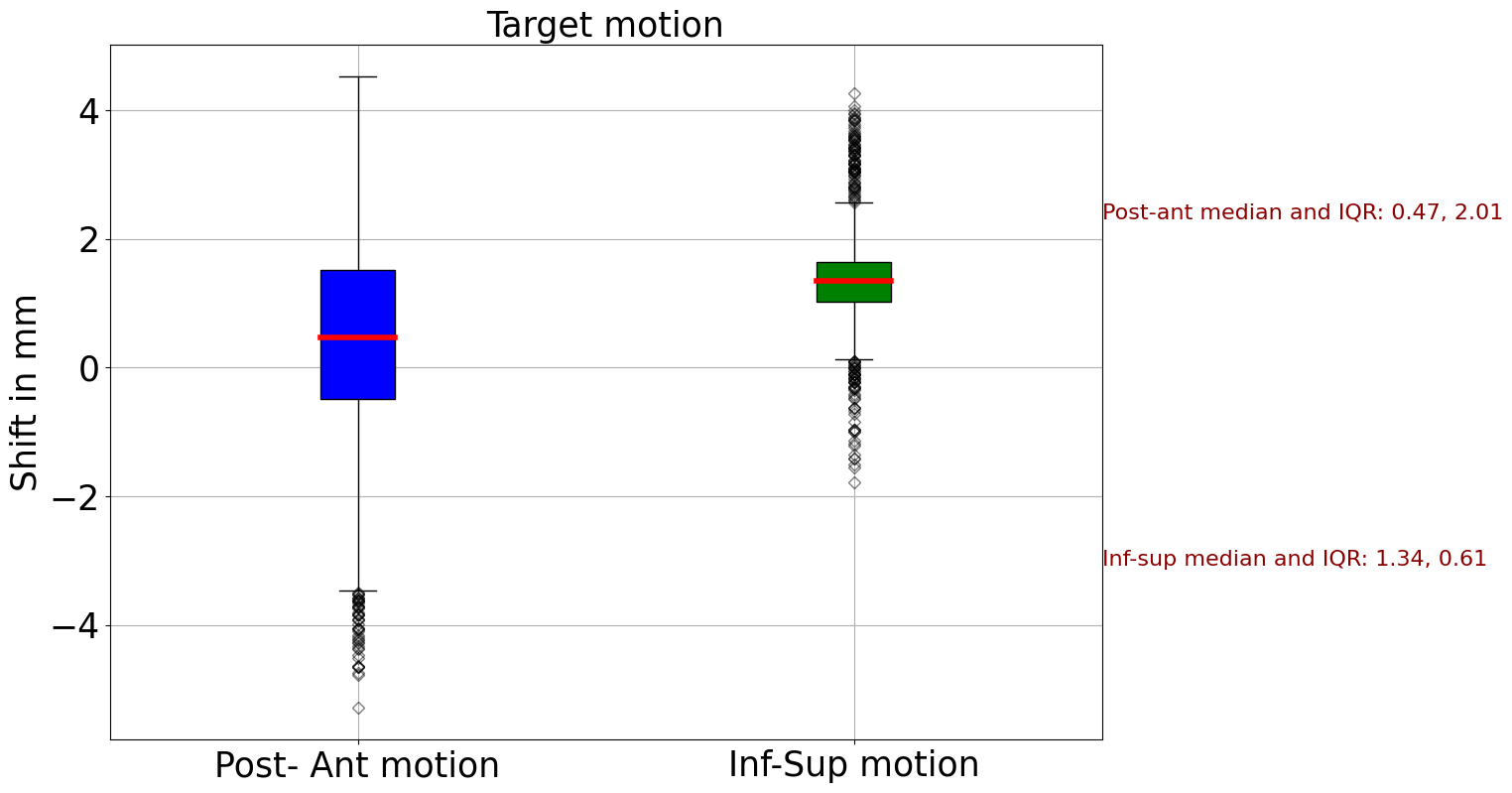

Supplement: Supplementary file 2 — Additional file 2: Box-plot of the primary tumour motion in posterior-anterior and inferior-superior directions for fractions 2-16. [file 13014_2022_2011_MOESM2_ESM.zip › 13014_2022_2011_MOESM2_ESM/S2-10.png]

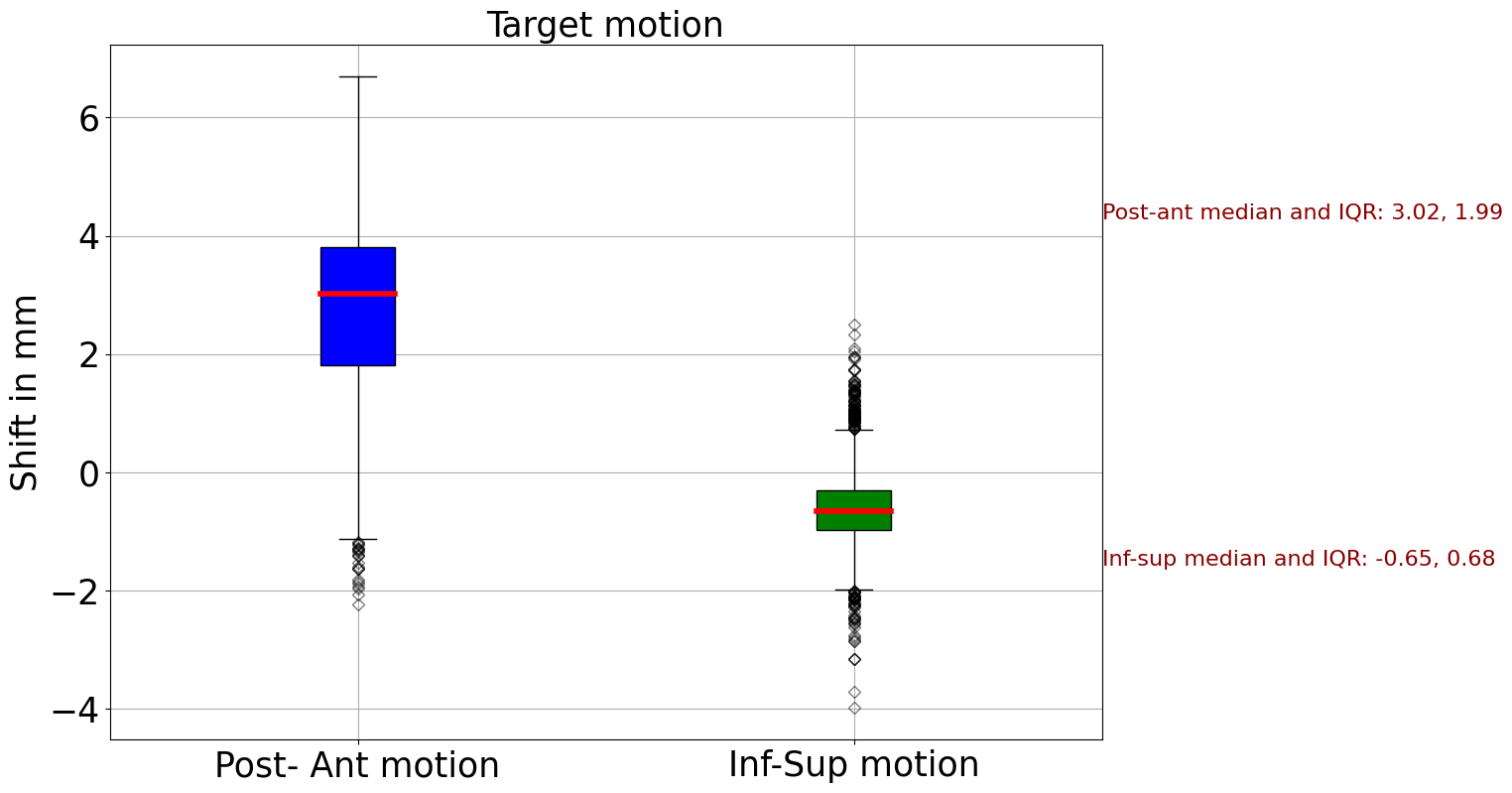

Supplement: Supplementary file 2 — Additional file 2: Box-plot of the primary tumour motion in posterior-anterior and inferior-superior directions for fractions 2-16. [file 13014_2022_2011_MOESM2_ESM.zip › 13014_2022_2011_MOESM2_ESM/S2-11.png]

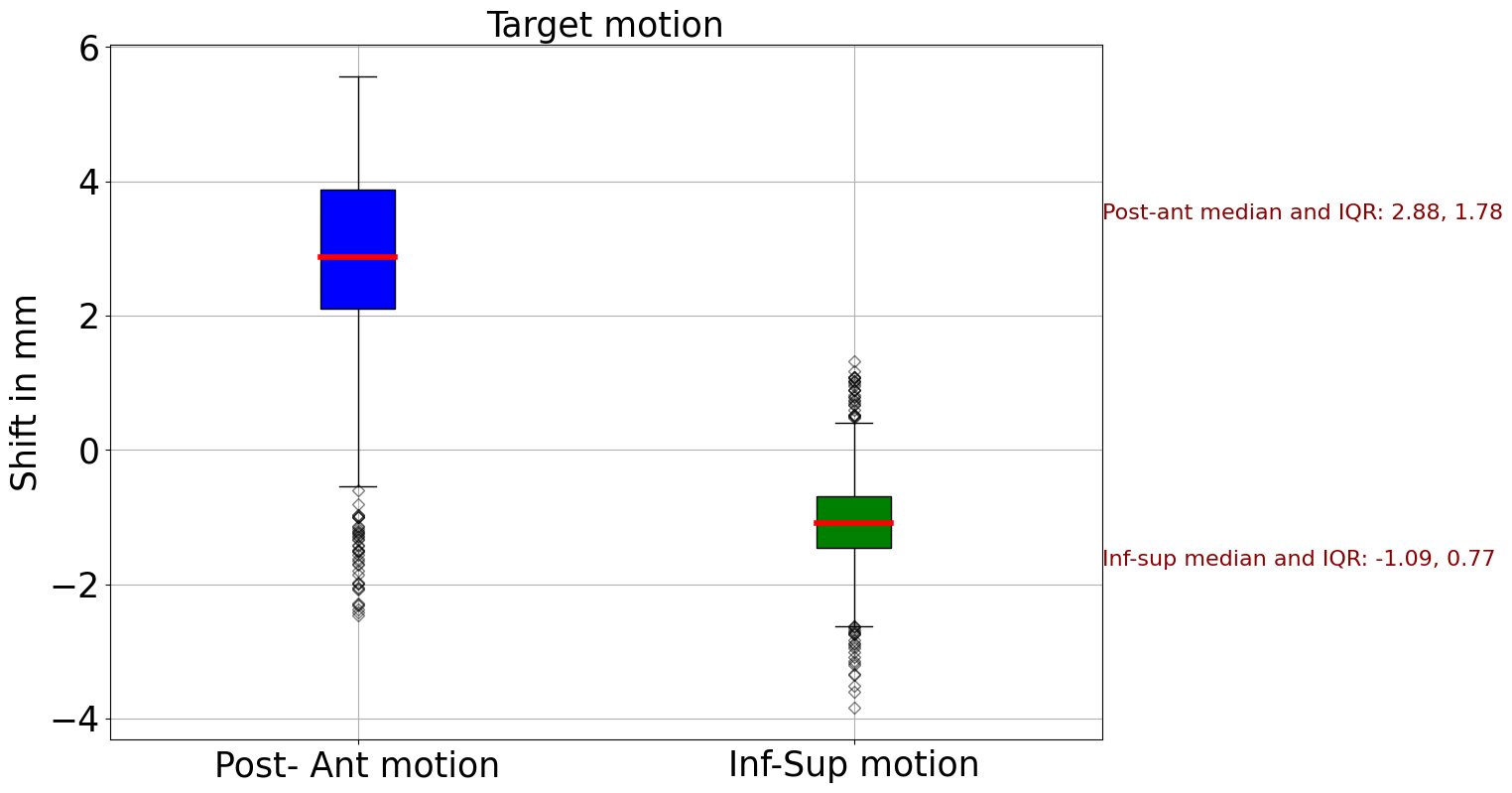

Supplement: Supplementary file 2 — Additional file 2: Box-plot of the primary tumour motion in posterior-anterior and inferior-superior directions for fractions 2-16. [file 13014_2022_2011_MOESM2_ESM.zip › 13014_2022_2011_MOESM2_ESM/S2-12.png]

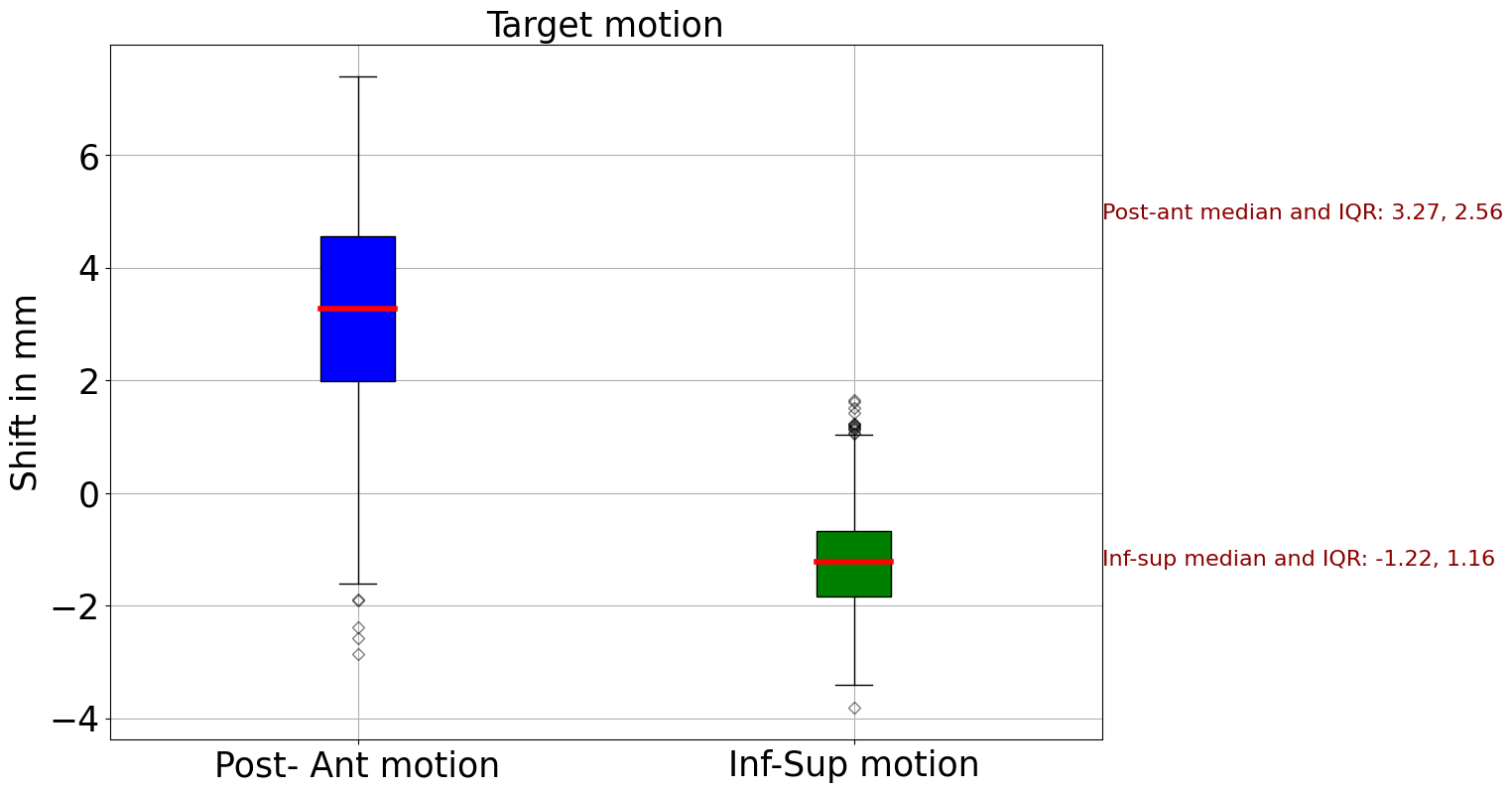

Supplement: Supplementary file 2 — Additional file 2: Box-plot of the primary tumour motion in posterior-anterior and inferior-superior directions for fractions 2-16. [file 13014_2022_2011_MOESM2_ESM.zip › 13014_2022_2011_MOESM2_ESM/S2-13.png]

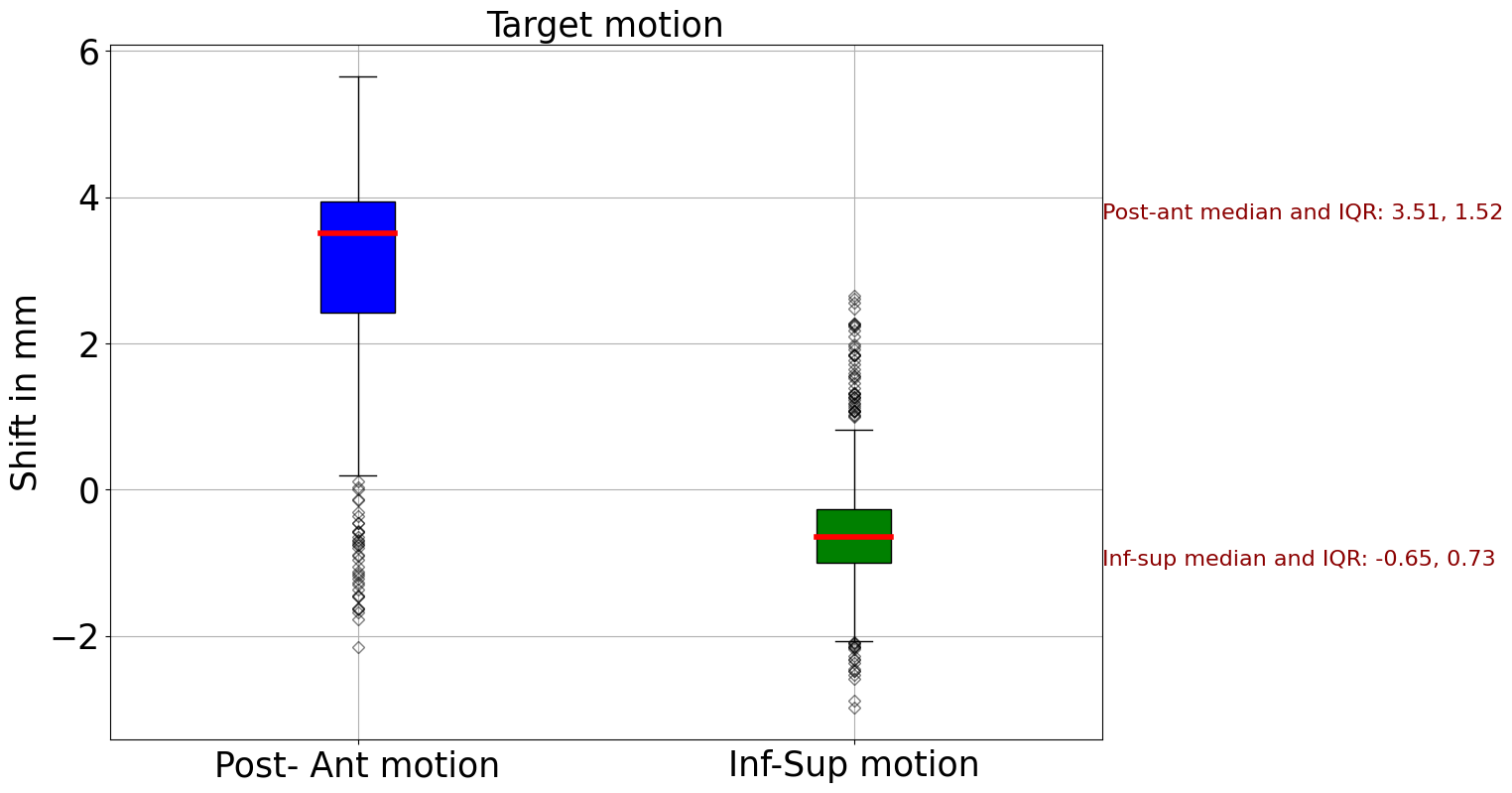

Supplement: Supplementary file 2 — Additional file 2: Box-plot of the primary tumour motion in posterior-anterior and inferior-superior directions for fractions 2-16. [file 13014_2022_2011_MOESM2_ESM.zip › 13014_2022_2011_MOESM2_ESM/S2-14.png]

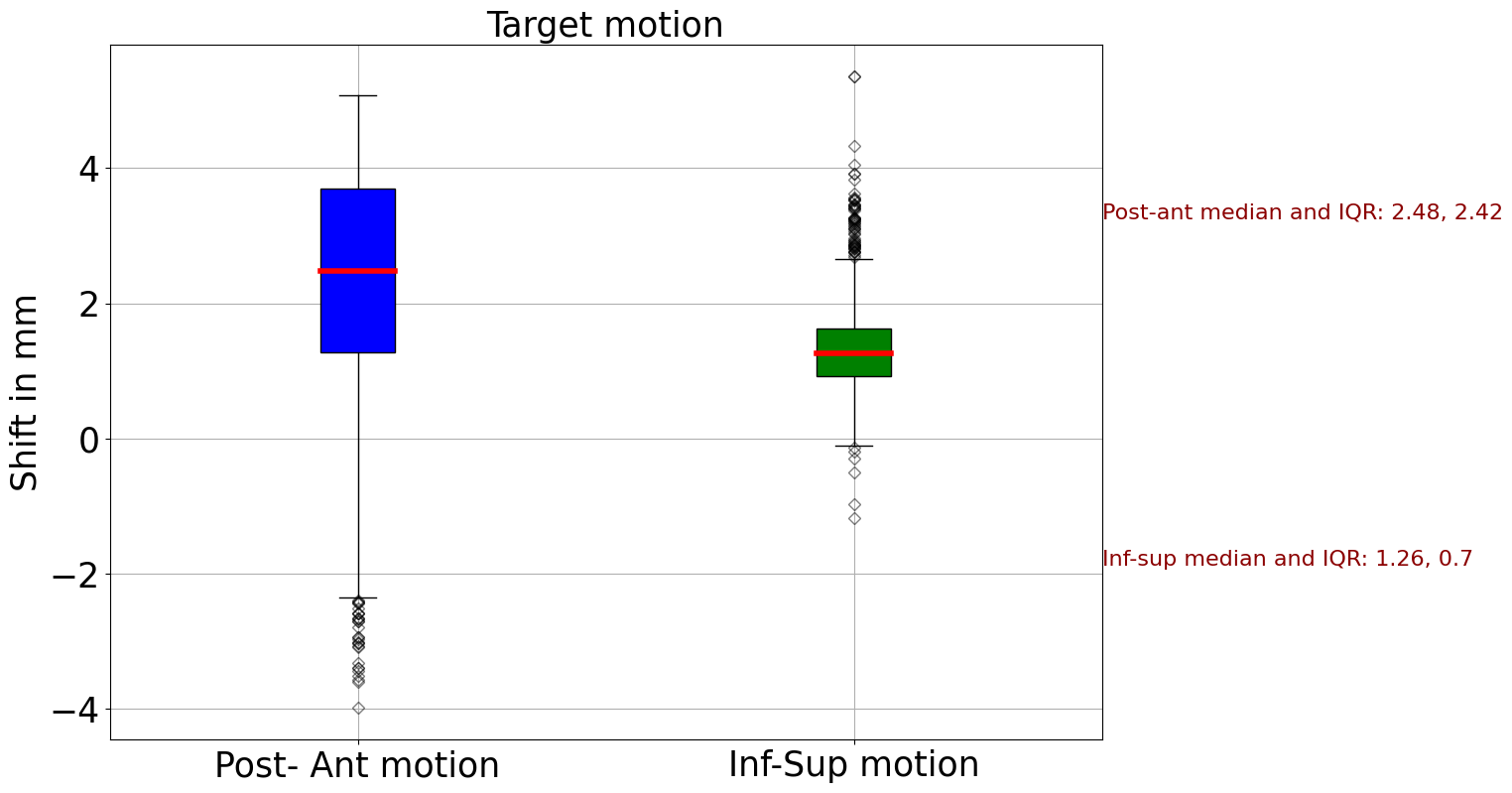

Supplement: Supplementary file 2 — Additional file 2: Box-plot of the primary tumour motion in posterior-anterior and inferior-superior directions for fractions 2-16. [file 13014_2022_2011_MOESM2_ESM.zip › 13014_2022_2011_MOESM2_ESM/S2-15.png]
